# Influence of Substrate Color on Oyster Shell Colonization

**DOI:** 10.17912/micropub.biology.001519

**Published:** 2025-04-21

**Authors:** Pauline Lawrence, Samantha Dishong, Elizabeth Hamman

**Affiliations:** 1 Biology, St. Mary's College of Maryland, Saint Marys City, Maryland, United States

## Abstract

This study investigated the influence of substrate color on the recruitment and colonization of *Crassostrea virginica* reef-associated organisms in an artificial reef system in the St. Mary’s River. Substrate color significantly affected community abundance, but the specific pattern depended on locomotion and species. The sessile community preferred blue substrate, which was largely driven by the strong settlement preference of tube-forming polychaetes (serpulid worms). Motile species showed recruitment preference for red shells. Mud coverage and erosion negatively affected the recruitment of sessile species but did not affect motile species recruitment.

**Figure 1. Functional-group specific substratum color discrimination in Crassostrea virginica reef-associated species:**
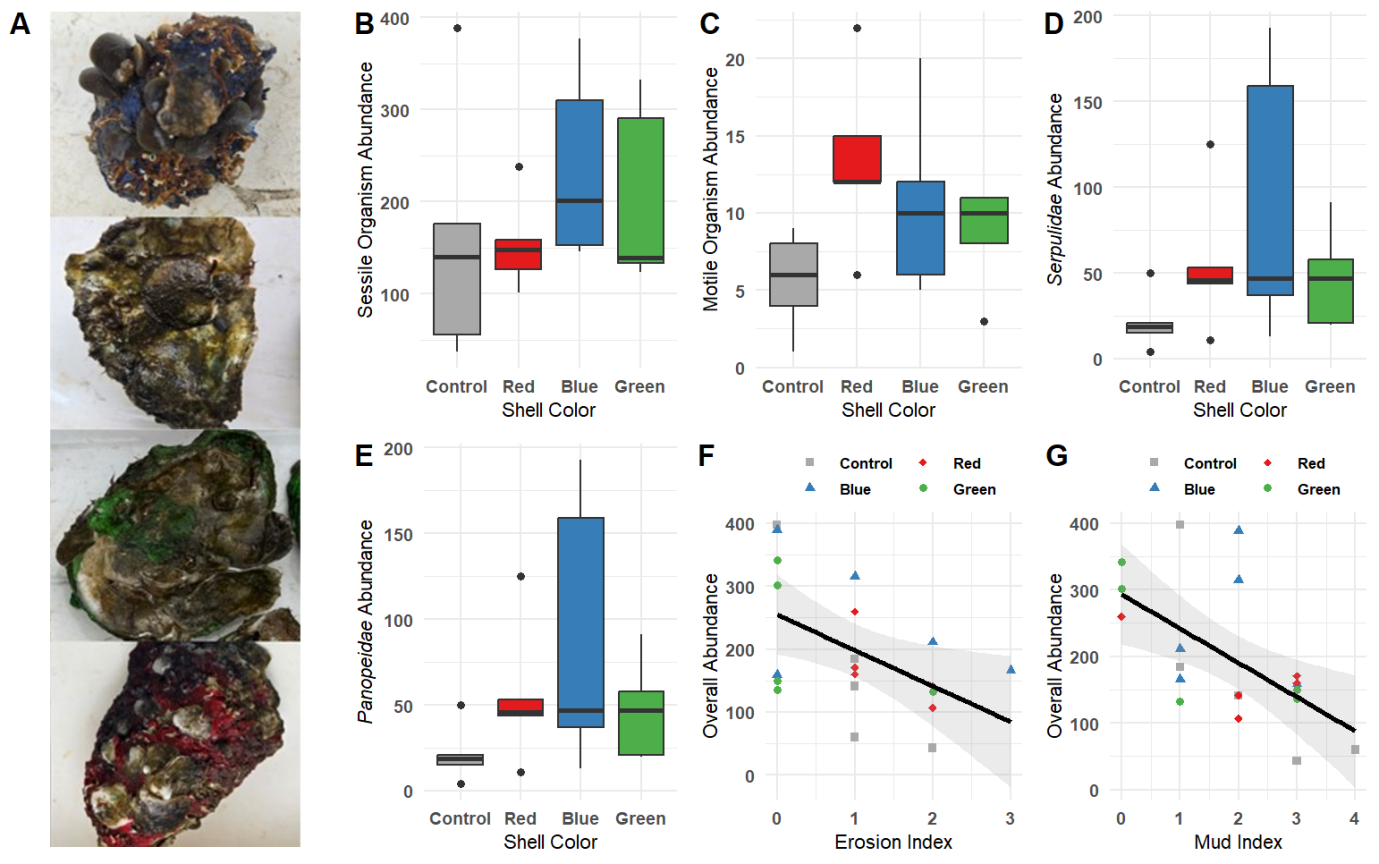
Panel **(A)** Photos of oyster shells from each treatment after 16 weeks in the water. Panels **B, C, D, and E** show boxplots of **(B)** sessile species (e.g., *C. virginica* spat, *Balanus spp., Brachidontin spp.,* and Serpulid worms, **(C)** motile organism counts (e.g., P *hyllodocid spp., Panopeid spp., C. sapidus, O. tau, C. bosquianus, G. strumosus, Palaemonetes pugio* , and *Hippocampus erectus* ), significant individual species: **(D)** Serpulid worms and **(E)** *Panopeid spp.* by shell color (control, red, blue, green). Panels **F and G** show the relationship between erosion (a measure of perforation/damage to new biological growth and/or shell) **(F)** and mud (a measure of mud coverage within the caged shell clusters) indices **(G)** on overall organism abundance, with point color indicating shell color

## Description

Oysters are a key foundation species in temperate estuaries that provide habitat and food web support for many economically and ecologically valuable species (Coen et al., 1999; Harding & Mann, 2000; Grabowski et al., 2005; Grabowski and Peterson, 2007; Jud & Layman, 2020). Processes such as colonization and recruitment assemble these diverse oyster reef communities over time as organisms gradually establish themselves in and around reef structures (Manley et al., 2010). The physical and biological characteristics of the habitat that later influence the persistence, distribution, and diversity of these communities also affect colonization and recruitment processes (Santiago et al., 2019).

In oyster reef communities, the accrual of habitat forming organisms (biological accumulation) and increased height above the benthos (vertical relief) are among the most important factors influencing the ability of an organism to recruit and persist within oyster structures (Soniat et al., 2004; Halon et al., 2018). Both vertical relief and biological accumulation increase the complexity of the habitat (Coen et al., 1999; Soniat et al., 2004; Halon et al., 2018), which is positively associated with increased biodiversity (Cunningham, 2023). While previous studies found effects of orientation (Soniat et al., 2004), material type (Strain et al., 2018), or physical heterogeneity (Halon et al., 2018) on benthic organism recruitment and growth, sensory factors, such as color, have largely been ignored.

Emerging evidence suggests that color may influence behavior, settlement, and community development (Mason et al., 2011; Roy et al., 2019; Cunningham, 2023). Even species with monochromatic vision may respond to specific wavelengths of light, and color variation may mimic variation in spectral cues provided by early settling microorganisms (e.g., eukaryotic algae) that are important to the settlement of habitat-structuring organisms (Mason et al., 2011; Rockwell et al., 2014; Cooney et al., 2024). Furthermore, in species with visual color detection, color preference may be influenced by their environment's complexity and learned associations (Roy et al., 2019). Since substrate color can vary spatially and temporally and can be altered by human activity (i.e., intentionally through use of alternative restoration materials such as brick or unintentionally through anthropogenic waste), it is also possible that substrate color can affect colonization and recruitment directly through the influence of phenotypic variation in camouflage strategies (Duarte et al., 2020) or by altering biological interactions between species in community structures (Satheesh & Wesley, 2010; Duarte et al., 2018; Cunningham, 2023). Given the potential for substratum color to affect how organisms interact with their environment and alter community assembly, it is important to investigate how substrate color influences the colonization and recruitment to communities such as that associated with *C.* *virginica* aggregations. This study investigated the role of substrate color on the processes of colonization and recruitment of reef-associated communities on *C. virginica* shells, focusing on how variation in substratum color settlement patterns and community development within artificially placed oyster shell clusters.

We placed oyster shells of four colors (red, blue, green, and natural) in cages on a restored oyster reef and monitored colonization after a 16-week deployment. We found a variety of organisms settled to shells of all four treatments (Panel A), and overall abundance varied by shell color. In particular, organism counts were 86.8% higher on blue compared to green (z = 4.47, p < 0.001) and 57.7% higher than the control (z = 3.44, p = 0.004, Table 1). This pattern was primarily driven by sessile species (Panel B), where the blue shells had 1.90 times higher sessile abundance than green and 1.57 times that of the control shells (Table 1). However, motile species exhibited a different pattern (Panel C). Pairwise comparisons of the resulting model revealed that the motile organism counts were 124.7% higher on red shells when compared to the control (z = 3.21, p = 0.007), while all other pairs were not significantly different with the inclusion of the erosion and mud indices along with the blocking term. Substrate color discrimination between these two groups could highlight the functional differences between the species in these groups. The motile species group primarily consists of small opportunistic predators or scavengers that need to both actively find food sources and avoid predation by larger predatory species. It is possible that red substrate selection provides increased camouflage while signaling an ample food supply.

Six of the 12 species had fewer than three observed individuals ( *C. sapidus, C. bosquianus, G. strumosus* , *O. tau, Hippocampus erectus, Palaemon spp.* ) and were not included in an individual analysis. Of the remaining species, three species ( *Phyllodocid spp., C. virginica* spat, and *Brachidontin spp.* ) did not show a preference for shell color (p > 0.05). Serpulid worm settlement to blue shells was 3.60 times higher than the control (z = 3.61, p = 0.002) and 2.91 times higher than green shells (z= 3.26, p = 0.006, Panel D, Table 1). Settlement preference for red shells was also observed. Red shell abundance was 3.07 times higher than the control (z = 3.00, p = 0.01) and 2.49 times higher than green shells (z = 2.61, p = 0.045, Table 1). *Balanus spp.* colonization of blue shells was 71.1% higher than green shells (z = 3.01, p = 0.014) and 81.6% higher than red shells (z = 3.72, p = 0.001), but there was no settlement preference observed between blue and the control shells (p > 0.05, Table 1). These findings are consistent with similar findings in which the barnacle species in the same genus ( *Balanus)* and tube-forming worms in the same order ( *Sabellariidae* ) as those in our study preferentially settled on red and blue substrates over yellow, white, and green (Satheesh & Wesley, 2010). The preferential settlement to red and blue substrates in multiple *Balanus* and *Sabellariidae* species could suggest that these colors attract microbial communities that are more attractive to these species or play a role in predator detection avoidance (Mason et al., 2011; Rockwell et al., 2014; Duarte et al., 2020; Cooney et al., 2024).

*Panopeid spp.* recruitment to red shells was 133.4% higher than the control (z= 2.72, p = 0.033). However, there appeared to be no recruitment preference between red shells and the remaining colors (blue and green, p > 0.05), which suggests that *Panopeid spp.* may prefer color in general over the natural color of *C. virginica* shells (Panel E, Table 1). Since the majority of the *Panopeid spp.* individuals found during our study were likely juveniles (≤ 20 mm), it is possible this finding is linked to their need for increased camouflage and crypsis during early life stages (Duarte et al., 2020).

Both mud and erosion reduced overall abundance (Panel F and G). As the erosion of the shell increased in a cage, the overall abundance of organisms decreased (z = -6.06, p < 0.001, Panel F). While we did not observe the source of erosion, damage occurred to both the shell and new growth such as serpulid worm tubes and *Balanus spp* . shells. Color did not affect the amount of erosion damage (p > 0.05). However, since the extent of the damage varied greatly among cages, other factors likely played a role in erosion damage occurrence. Increased mud coverage also reduced total abundance (z = -7.09, p < 0.001, Panel G). This finding is also unsurprising, considering previous studies have highlighted sedimentation as a threat to the survival of sessile species (Soniat et al., 2004). Both environmental variable impacts were largely experienced by sessile species, as neither had a significant effect on motile species abundance alone (p > 0.05).

Overall, this study provided evidence that substrate color may serve as a factor that influences the colonization and recruitment of oyster shells. Functional-group-specific differences in substratum color selection suggest that there may be a link between substrate color selection and the functional needs of the organism. However, this study was limited to 16 weeks of settlement in a small restored reef. Future studies could investigate how color influences community development across greater spatial and temporal scales to examine how these relationships change over time and how they may vary in other benthic habitats within the Chesapeake Bay. The findings of this study could also be explored within the context of restoration efforts as a potential means to increase habitat-forming organism abundance through the intentional coloration of restoration materials. Additionally, since mud prevalence significantly impacted the sessile community, future research could employ methods such as cage stilts or suspension to reduce variability in sedimentation.

**Table 1.** Output from Emmeans post hoc tests for individual species with significant p-values highlighted in green. The degrees of freedom column was removed for clarity due to emmeans utilizing z-tests.

**Table:** 

**Species**	**contrast**	**estimate**	**SE**	**z.ratio**	**p.value**
***Panopeid spp.***	Red - Control	0.8496	0.3128	-2.7159	0.0334
	Blue - Control	0.7376	0.3204	-2.3020	0.0975
	Green - Control	0.4983	0.3495	-1.4257	0.4832
	Red - Blue	0.1120	0.2356	0.4754	0.9645
	Red - Green	0.3513	0.2885	1.2176	0.6156
	Blue - Green	0.2393	0.2915	0.8211	0.8445
***Phyllodocid spp.***	Red - Control	0.7115	0.4182	-1.7013	0.3229
	Blue - Control	0.3485	0.4373	-0.7969	0.8559
	Green - Control	-0.6085	0.5508	1.1047	0.6866
	Red - Blue	0.3630	0.3656	0.9928	0.7535
	Red - Green	1.3200	0.5207	2.5351	0.0547
	Blue - Green	0.9570	0.5293	1.8079	0.2695
***C. virginica spat***	Red - Control	0.2789	0.2430	-1.1480	0.6597
	Blue - Control	0.5148	0.2297	-2.2407	0.1124
	Green - Control	0.1011	0.2012	-0.5024	0.9585
	Blue - Red	0.2359	0.1930	-1.2222	0.6127
	Red - Green	0.1778	0.2346	0.7580	0.8732
	Blue - Green	0.4137	0.2294	1.8032	0.2718
***Brachidontin spp.***	Red - Control	0.3491	0.4810	-0.7258	0.8868
	Blue - Control	0.3362	0.4837	-0.6952	0.8990
	Green - Control	-0.9478	0.6465	1.4660	0.4582
	Red - Blue	0.0129	0.4970	0.0259	1.0000
	Red - Green	1.2970	0.5952	2.1792	0.1290
	Blue - Green	1.2841	0.5408	2.3743	0.0821
**serpulid worms**	Red - Control	1.1220	0.3736	-3.0032	0.0142
	Blue - Control	1.2789	0.3539	-3.6142	0.0017
	Green - Control	0.2104	0.3722	-0.5653	0.9424
	Blue - Red	0.1569	0.2768	-0.5669	0.9419
	Red - Green	0.9116	0.3494	2.6093	0.0449
	Blue - Green	1.0685	0.3274	3.2635	0.0061
***Balanus spp.***	Red - Control	-0.2367	0.1780	1.3300	0.5437
	Blue - Control	0.3599	0.1659	-2.1700	0.1316
	Green - Control	-0.1771	0.1481	1.1962	0.6293
	Blue - Red	0.5966	0.1605	-3.7165	0.0012
	Red - Green	-0.0596	0.2037	-0.2926	0.9913
	Blue - Green	0.5370	0.1784	3.0105	0.0139

## Methods

This research was carried out within a restored oyster reef in the St. Mary’s River, a tributary of the Chesapeake Bay. We sorted 400 shells into four treatment groups of 100 each: red, blue, green, and control. We painted shells according to their treatment group color using water-resistant non-toxic acrylic paint (ABEIER _Ⓡ_ , Cixi, Zhejiang, China) and left control shells unpainted. Subsequently, we coated all shells, including the control, with a clear, matte top coat spray paint (Rust-Oleum 2X ultra cover spray paint in “matte clear”, Rust-Oleum Corporation, Vernon Hills, Illinois) to seal the acrylic paint and control for any effects related to the paint on the non-colored control group. For cage construction, we zip-tied squares of 2.54 cm and 1.27 cm metal mesh together to form 25 x 25 cm cubes. Each treatment group had five replicates with 20 shells in each cage. We deployed our experiment in a randomized block design. We placed each cage approximately 2 meters apart in an 8 x 6 meter zone. Cages were then left undisturbed for 16 weeks (early June to late September) within the study site.

After the 16-week study period, we removed the cages from the river and immediately placed them inside individual tubs to prevent organism loss during transport to shore. We examined individual shells in each cage and recorded all the species and number of organisms from each cage. Due to observed variation in mud coverage and signs of erosion, we assigned each cage an index number for mud coverage (0-4, with 4 being the highest mud coverage) and erosion (0-3, with 3 being the highest level of observed damage).

We analyzed the effect of color on colonization using generalized linear models with a negative binomial distribution. Substrate color, erosion, and mud level were included as fixed effects, and block as a random effect. We validated all models using the DHARMa package (Hartig, 2024). We used Emmean’s post hoc tests in the Emmeans package (Lenth, 2024). All analyses were conducted in R Version 4.4.2 (“Pile of Leaves”, R Core Team, 2024).
